# Developing a Valuation Function for the Preference-Based Multiple Sclerosis Index: Comparison of Standard Gamble and Rating Scale

**DOI:** 10.1371/journal.pone.0151905

**Published:** 2016-04-28

**Authors:** Ayse Kuspinar, Simon Pickard, Nancy E. Mayo

**Affiliations:** 1 School of Physical and Occupational Therapy, Faculty of Medicine, McGill University, Montreal, QC, Canada; 2 Center for Pharmacoepidemiology and Pharmacoeconomic Research and Department of Pharmacy Systems, Outcomes and Policy, University of Illinois at Chicago, Chicago, Illinois, United States of America; 3 Division of Clinical Epidemiology, McGill University Health Center, Montreal, QC, Canada; Corvinus University of Budapest, HUNGARY

## Abstract

**Objective:**

The standard gamble (SG) and rating scale (RS) are two approaches that can be employed to elicit health state preferences from patients in order to inform decision making. The objectives of this study were: (i) to contribute evidence towards the similarities and differences in the SG and the RS to reflect patient preferences, and (ii) to develop a multi-attribute utility function (MAUF) (i.e., scoring algorithm) for the PBMSI.

**Study Design:**

Two samples were recruited for the study. The first sample provided cross-sectional data to generate the preference weights which were then used to develop (_D_) the MAUF_D_. The distribution of SG and RS were compared across levels of perceived difficulty. The second sample provided additional data to validate (_V_) the MAUF, termed MAUF_V_.

**Results:**

The mean RS values ranged from 0.39 to 0.65, whereas the mean SG values were much higher ranging from 0.80 to 0.91. Correlations between the two methods were very low ranging from -0.29 to 0.15. Bland-Altman plots revealed the extent of differences in values produced by the two methods.

**Conclusion:**

In contemplating trade-offs in the selection of a preference-based elicitation approach for a MAUF that could guide clinical decision making, results suggest the RS is preferable in terms of feasibility and validity for MS patients. The PBMSI with patient preferences shows promise as a measure of health-related quality of life for MS.

## Background

Multiple Sclerosis (MS) is a progressive, demyelinating disease of the central nervous system (CNS) that affects all aspects of an individual’s life. MS produces a range of unpleasant and debilitating symptoms, including fatigue, muscle weakness, loss of memory and concentration, to name a few. These can have a profound impact on daily functioning, relationships, and social and leisure activities.

Health-related quality of life (HRQL) is a multi-dimensional outcome, represented by physical, mental and social well-being. HRQL measures are frequently used to evaluate rehabilitation interventions. This is especially true for chronic conditions like MS, as the management of these diseases are rehabilitative in nature, rather than curative. A recent systematic review in MS[1] identified that HRQL was an important and common outcome in clinical trials of exercise, self-management and cognitive-behavioural therapy.

One approach to assessing HRQL is through the use of health profiles. Examples of generic health profiles include the generic Short Form-36 (SF-36)[2] and disease specific health profiles include the MS Quality of Life-54 (MSQOL-54)[3], which are both scored by sub-scale. A challenge with using health profiles in clinical trials is that if a treatment has a positive effect on physical health but a negative one on mental health, it is impossible to determine whether the intervention resulted in a net improvement or decline in HRQL.[1]

Another approach to measuring HRQL is through the use of preference-based measures. Examples of such measures include the Euro-QoL-5 Dimension (EQ-5D)[4] and the Health Utilities Index Mark 3 (HUI3)[5]. These measures not only provide descriptive information on the various dimensions of health, but also provide a value for each one. They have the advantage of leading to a single number (generally from 0 to 1) that balances gains in one domain against losses in another.

A feature of all these measures is that they are generic, and the preference weights are obtained by asking members of the general population to consider the health-impact of each item, whether or not they have experienced the effect. Although general population weights are important for economic evaluation, they have little relevance in clinical research and decision-making. When making clinical decisions about which treatment is better or worse for a given patient, the patient’s perspective on the benefits and risks is important. Patient’ preferences for health states have been shown to differ systematically from those obtained from the general population[6], with patients valuing sub-optimal health states higher. Furthermore, the psychometric properties of these generic preference-based measures in MS have recently been reviewed and limitations identified[7].

There are two steps involved in developing a preference-based measure. The first step is to develop a classification system with items and response options. The second step involves asking patients or the general population to indicate their preferences for (or how much they value) each of the items in the classification system, using one or more standard techniques. These preferences are then combined in a scoring algorithm, also known as a multi-attribute utility function (MAUF), to provide a score from 0 (dead) to 1 (perfect health) for any individual who completes the questionnaire.

Two of the most well-known methods of valuing health states are the standard gamble (SG) and the rating scale (RS).[8] The RS typically asks individuals to place a given state on a vertical ruler-like scale (i.e. feeling thermometer). With the SG, respondents are asked to indicate the extent to which they would risk dying (with a treatment) that can return them to full health. To date, no agreement has been reached in terms of which method should be used in the valuation of health states. Table 1 summarizes the key characteristics associated with each method. There are such strong conceptual differences between the two methods that could affect patients’ capacity to understand and respond appropriately to the task demanded, a head-to-head comparison was thought to be of use in the context of MS and in the context of developing a preference based measure.

**Table 1:** Summary of standard gamble and rating scale.

Standard Gamble	Rating Scale
• Respondents are asked to indicate the extent to which they are willing to risk death for an improvement in health	• Respondents are asked to place a given health state on a vertical ruler-like scale (i.e. feeling thermometer).
• Based on the axioms of utility theory of Von Neumann and Morgenstern.	• Based on psychometric or measurement theory
• Includes element of risk	• Does not include element of risk
• Associated with cognitive burden	• Simple and easy to use
• Prone to risk aversion bias	• Prone to response spreading

A MS specific classification system, titled the Preference-Based Multiple Sclerosis Index (PBMSI), was recently developed based on semi-structured interviews from 185 patients with MS [9–11]. The PBMSI classification system consists of 5 items with 3 response levels per item, producing 243 (3^5^) different health states, or combination of responses. The aim of this paper is to complete the PBMSI by asking patients with MS to indicate their preferences for the different items in the classification system.

Therefore, the objectives of this study were: (i) to contribute evidence towards the similarities and differences in the SG and the RS to reflect patient preferences, where contrasts were on absolute values and level of difficulty, and (ii) to develop a MAUF (scoring algorithm) for the PBMSI.

## Methods

**Fig 1:**
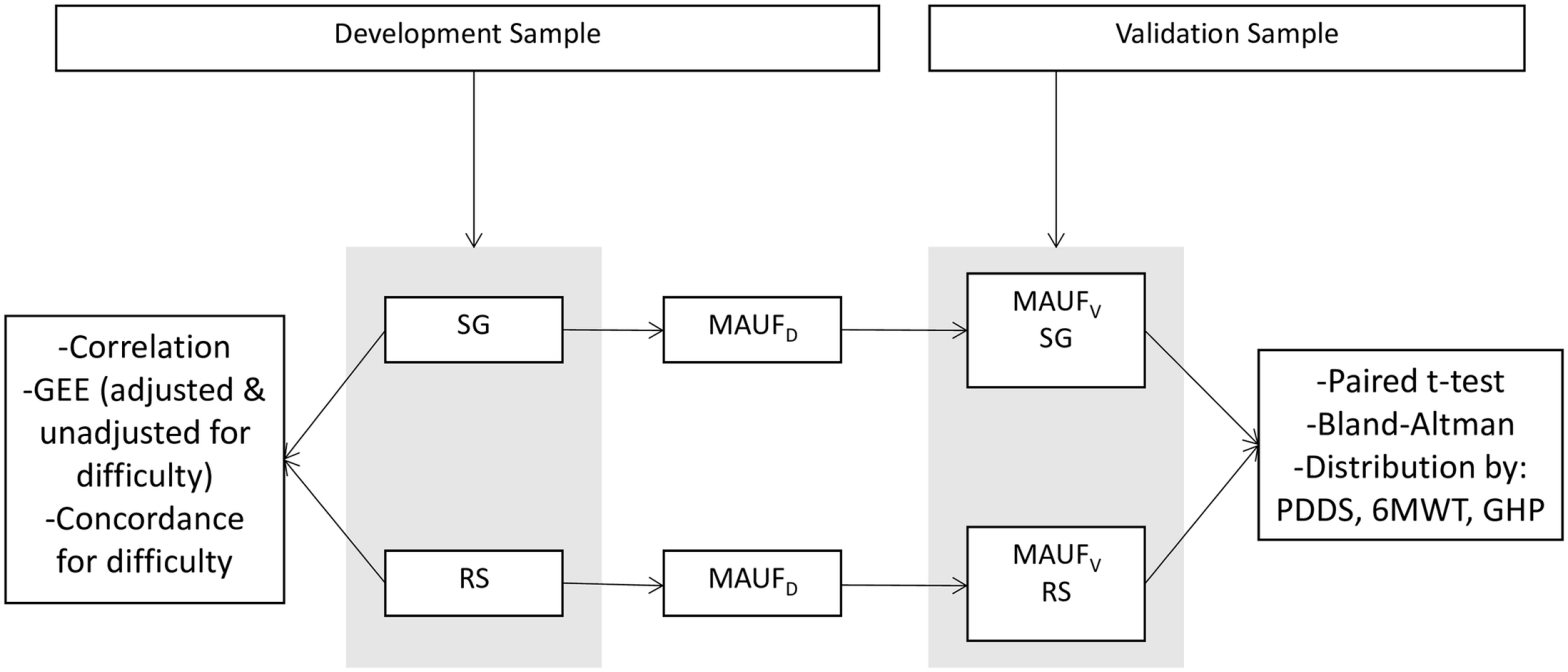
A flow diagram of the methodological steps involved in the study.

Fig 1 presents the methodological steps for this study. Two different samples were recruited. The first (development) sample provided cross-sectional data to generate the preference weights for the valuation of health-states which were then used to develop (_D_) the MAUF_D_. For the development sample, the distribution of SG and RS were compared across levels of perceived difficulty in completing the valuation. The next step was to produce the MAUF_D_ based on valuations obtained from both the SG and RS. The second sample provided additional cross-sectional data to validate (_V_) the MAUF, termed MAUF_V_. The parameters from the MAUF_D_ were applied to the validation sample to produce the MAUF_V_ and the distribution compared across key measures known to reflect the impact of MS.

### Selection of Subjects

The development sample for the valuation of health states was recruited through advertising in three venues: MS Society of Canada website; the 2012 Quebec Summit on Multiple Sclerosis; and outpatient MS clinic of the Montreal Neurological Hospital. To participate, individuals had to be diagnosed with MS and be older than 18 years of age. The study was approved by the McGill University Health Center Research Ethics Board and written informed consent was obtained from participants prior to doing the online survey.

The validation sample was subjects with MS who were participating in a clinical trial of exercise (ClinicalTrials.gov; Registration Number: NCT01611987). The protocol for this study has been published.[12] Briefly, participants were recruited from 3 MS clinics in the Montreal area and were aged 19–65, diagnosed after 1994, ambulatory, and able to speak and read English or French. Participants were excluded if they had an additional illness that restricted their function, had suffered at least one relapse during the past 30 days, or were unable to respond to simple questions on orientation and memory. This sample was ideal for the assessment as they were stable at time of recruitment and had the language and cognitive capacity to understand the questions. The ethics committees of each participating hospital: (i) McGill University Health Center Research Ethics Board, (ii) Clinique Neuro-Rive Sud Research Ethics Board, and (iii) Centre Hospitalier de l'Université de Montréal Research Ethics Committee, approved the study.

### Measures

The main measure for this study was the PBMSI, administered to both the development and validation samples. Two methods of valuing the health states from the PBMSI were the SG and RS used to derive MAUF_D_. Measures of global disability, walking capacity and general health perception were used to validate MAUF_V_.

#### PBMSI

The PBMSI is a brief self-administered questionnaire consisting of five items: walking, fatigue, mood, concentration, and roles and responsibilities. Each item has three response options, and the recall time frame is ‘over the past week’. The classification system produces 243 (3^5^) health states.

#### Selection of health states for valuation

Each patient valued 12 health states: 5-single attribute level states, 5 corner states, all worst and all intermediate states. These states are as follows:

- Single-attribute level states: a given item was described at less than full function (response level 2) while all other items were set at their best level (response level 1).
- Corner states: a given item was described at its worst level (response level 3) while all other items were set at their best level (response level 1).
- All worst was described as the worst level on all items (response level 3), and all intermediate was described as less than full function on all items (response level 2). Patients also assigned a value for the state ‘dead’ on the RS. A value for the state ‘dead’ was not required for the SG, as it was anchored from dead to perfect health.

Preferences for the above health states were obtained from patients with MS using an online survey. In the survey, patients were asked to fill out the PBMSI and answer certain socio-demographic and clinical questions. Then they were asked to value selected health states using the SG and RS.

#### Standard gamble

Patients were asked to rate the single-attribute and corner states using the SG (SG). In the SG, patients were presented with a less than perfect health state (i.e. a corner state or single-attribute state), and asked to imagine themselves in that health state for the rest of their life. Then they were asked to imagine that they were given a treatment. If the treatment was successful, they would be restored to full health. But if the treatment were to fail, they have a probability of dying immediately. Essentially respondents are asked to indicate the highest risk of death (in percentage) they would accept with the treatment. However, questionnaire that elicited these probabilities, referred to death as “failure”. This is a common procedure in the literature.[13–17] The response options are given in a drop down menu, as follows: ‘0% chance of ‘failure’ (100% chance of ‘success’)…5% chance of ‘failure’ (95% chance of ‘success’)…etc.’ Patients were asked to select only one response option from the list provided. The probability of ‘success’ that they were willing to accept with the treatment was their SG value (i.e. 100% ‘success’ is equal to a SG value of 1.0, 95% ‘success’ is equal to a SG value of 0.95 etc.)

The format also allowed for the assessment of states worse than dead if respondents indicated that they would take the treatment even if it had 0% chance of ‘success’ (100% chance of ‘failure’).

#### Rating scale

Patients were asked to rate each of the single-attribute and corner states on a RS from 0 to 100, where zero was the worst imaginable health state and 100 was the best imaginable health state. Patients were also asked to provide on the RS a value for the state ‘dead’. If state dead was identified as the worst state and was placed at the 0 end of the scale, then preferences were simply equal to the scale value given to each health state. If death was not identified as the worst state but was placed on some intermediate point on the scale (*d*), then preferences were measured as: *(x-d)/(1-d)*, where *x* was the rating given to a health state and *d* was the rating given to death.

#### Difficulty

At the end of the survey patients were asked to rate how difficult it was to answer the PBMSI items, the RS, and the SG. Responses were recorded on a four-point Likert scale (very easy, fairly easy, fairly difficult, and very difficult).

#### Global disability

Global disability was measured using Patient-Determined Disease Steps (PDDS), self-reported outcome of disability in MS.[18] It has nine ordinal levels ranging between 0 (normal) and 8 (Bedridden) and PDDS scores can be converted into classifications of mild, moderate, or severe disability.[19] The PDDS is a surrogate measure of the Expanded Disability Status Scale (EDSS) and has shown to be strongly correlated with the EDSS.[20]

#### Functional exercise capacity

The 6-Minute Walk Test is a simple performance-based test that measures functional exercise capacity. The reliability of the 6-Minute Walk Test has been assessed in persons with MS. The intra-class correlation coefficient is 0.96 for test-retest reliability and 0.93 for inter-rater reliability.[21]

#### General health perception

The first question of the RAND-36 measures general health perception, and formulated as, “In general, would you say your health is…,” with five nominal response options ranging from excellent to poor.[22] General health perception is easy to measure and can provide information on the person’s well-being and overall HRQL. Furthermore, it has been shown to be a predictive factor in the progression of disease.[23,24]

#### EQ-5D

The EQ-5D[25] is a generic preference-based measure of HRQL that consists of two parts. The first part includes 5 separate domains; mobility, self-care, usual activities, pain/discomfort and anxiety/depression. Each domain has 3 levels: no problems, some problems, extreme problems.[25] The MAUF_D_ was compared against the EQ-5D, as it is a commonly used preference-based measure in MS and is recommended by the National Institute for Health and Care Excellence for economic evaluation.

### Statistical Methods

For the development sample, the distribution of SG and RS values was obtained for each health state and plotted by quartile; Pearson correlation coefficients were also calculated.

Concordance between the reported levels of difficulty for the SG and RS was presented and agreement assessed using un-weighted and weighted and Kappa. Generalized estimating equations were used to assess the impact that reported level of difficulty had on SG and RS values, considering the correlation arising from multiple valuations per person.

Two MAUF (i.e. scoring algorithms) were developed (MAUF_D_): one based on SG values and the other based on RS values. The methodology used to develop the MAUF_D_ closely followed the procedures described in the manual for the development of the HUI3.[26]

The person-mean approach was used to develop the valuation functions.[26] In other words, the functions were estimated from the mean responses of the sample for the single-attribute health states and corner states.

A utility scale runs from 0.0 (dead) to 1.0 (all best/perfect health). Disutility equals one minus utility (disutility = 1 –utility). Thus, the disutility scale ranges from 0.0 for all best/perfect health to 1.0 for dead.

If the sum of the disutility corner states is equal to 1.0, then the valuation function is additive. However, if the sum of the corner states is not equal to one, then the valuation function is multiplicative. The multiplicative function, as specified by MAUT was:

$$u^{,}=(1/c)\left[\prod_{j=1}^{n}\left(1+c*c_{j}*u_{j}^{,}\right)-1\right]$$ (1)

where, *u*′ is the required disutility of any PBMSI health state on the perfect health = 0.0, dead = 1.0 scale; *j* is the number of PBMSI items which was 5; *c*_*j*_ is the person-mean disutility for the corner state; $u_{j}^{i}$ is single-attribute level disutility score; and $\prod_{j=1}^{n}$ is the product of all. $\left(1+c*c_{j}*u_{j}^{\prime }\right)$. The scaling parameter *c* was calculated by iteratively solving the following equation:

$$1+c-\left[\prod_{j=1}^{5}\left(1+c*c_{j}\right)\right]=0$$ (2)

where $\prod_{j=1}^{5}$ is the product of all (1+c*c_*j*_)from *c*_1_ to *c*_5_; and *c*_*j*_ is the person-mean disutility for the corner state.

The scaling parameter c depends on the sum of the corner disutility states:

$$\text{If}\;\;\overset{5}{\underset{j=1}{\sum }}c_{j}>1\;\text{ then }-1\;<c<\;0;$$ (3a)

$$\text{if }\overset{5}{\underset{j=1}{\sum }}c_{j}=1\;\text{ then }c=\;0,\text{ and the valuation function is additive};$$ (3b)

$$\text{and if}\overset{5}{\underset{j=1}{\sum }}c_{j}<\;1\;\text{then }c>\;0.$$ (3c)

If the valuation function is additive, *c* = 0 is the only root of Eq 2. If the valuation function is not additive, Eq 2 will have 2 roots: (i) a trivial solution (*c* = 0) and (ii) a non-trivial solution (*c* ≠ 0). We will be searching for the non-trivial solution, and the sum of the corner states will tell us where to search for it (i.e. if sum of corner states is greater than 1, then -1 < *c* < 0; if sum of corner states is less than 1, then *c* > 0).

Excel Solver was used to iteratively solve for the scaling parameter *c*. All other analyses were conducted using SAS9.3.

We estimated the sample size for this valuation to yield a 95% confidence interval (95%CI) around the mean value for the SG and RS of ± 0.05 points. Clinically meaningful difference on the SG (as well as the RS) is approximately 0.10 points[27]; half the difference was chosen as it would not be meaningful and, therefore, this CI would indicate precision in the estimates of value.

Calculation of the 95% CI requires an estimate of the population standard deviation (SD). To our knowledge, there are no studies have reported the SD for the SG in people with MS. Therefore, sample size calculations were based on the values obtained for the RS in the MS Life-Impact Study[9, 10, 28] conducted in a similar population. The SD of the RS value for ‘best imaginable health’ was 0.08. Based on this information the number of people required per health state was equal to 10 (calculated using the following formula: 1.96*(0.08n)=0.05). As there were 5 corner states, the required sample size for this study was 50 people.

Agreement between the SG MAUF and RS MAUF for both samples was depicted using scatter plots. For perfect agreement, all data points are expected to be on the diagonal line, the line of equality. For both the development and the validation samples, the Bland-Altman method was used to analyze agreement between the SG MAUF and the RS MAUF. This method contrasts the mean difference between two MAUF (y axis) against the average of the two MAUF, which represents the latent trait of “utility”. The graph shows 95% limits of agreement around the mean difference (1.96 SD). Perfect agreement between the SG MAUF and the RS MAUF would be indicated by a mean difference equal to 0 and no pattern across the latent trait. A paired t-test was used to contrast values between the MAUF SG and MAUF RS.

The distribution of items on the PBMSI obtained from the clinical trial validation sample was identified. The known-groups method was used to test the discriminative ability of the standard gamble and RS MAUF_V_ against different measures of disability, namely the PDDS, the 6MWT and the general health perception item of the RAND-36. The MAUF_V_ was also compared against the generic preference-based measure EQ-5D. The linear test for trend was employed to test if gradients across levels of disability was statistically significant.

## Results

### Sample

Table 2 presents the demographic and clinical characteristics of the two samples, development and validation. These samples were chosen using quite different sampling frames, and hence were expected to differ somewhat. However, the two samples were similar on age (mean ~ 47 years) and proportion women (75%-79%). The clinical trial (validation) sample was comprised of people recruited into an exercise intervention trial and showed lower disability in walking (level 1), lower fatigue, better mood, but more challenges with regular roles and responsibilities. Also shown is the number of people in the most common health states. For example, 2% of the development sample and 8% of the validation sample had the health state 11111, reflecting the best level on all 5 dimensions. Furthermore, approximately 13% of the samples had the health state 22111, reflecting some problems with walking and fatigue, but no problems with mood, concentration, and roles and responsibilities. No statistical comparison between samples was done because it was known from the outset that these two samples did not arise from the same population.

**Table 2:** Demographic and clinical characteristics of the Development and the Validation sample.

Characteristics	Mean (SD) or N (%)	
	Development Sample	Validation Sample
Age (y)	46.6 (11.5)	47.3 (9.97)
Women / Men	48 / 13 (79 / 21)	48 / 16 (75 / 25)
English/French*	44 / 17 (72 / 28)	14 / 50 (22 / 78)
University/College/High School	36 / 17 / 8 (59 / 28 / 13)	47 / 13 / 4 (73 / 20 / 6)
VAS health state (0–100)	66.1 (16.4)	73.0 (14.0)
PBMSI Health State		
11111	1 (2)	6 (8)
12121	5 (8)	4 (6)
12221	6 (10)	5 (8)
22111	8 (13)	9 (14)
22222	8 (13)	3 (5)
Other	33 (54)	37 (58)
Walking		
1	23 (38)	29 (48)
2	29 (48)	30 (49)
3	9 (15)	2 (3)
Fatigue		
1	10 (16)	20 (33)
2	49 (80)	35 (57)
3	2 (3)	6 (10)
Mood		
1	29 (48)	37 (61)
2	30 (49)	22 (36)
3	2 (3)	2 (3)
Concentration		
1	20 (33)	28 (44)
2	35 (57)	34 (54)
3	6 (10)	1 (2)
Roles & Responsibilities		
1	37 (61)	19 (31)
2	21 (34)	42 (68)
3	3 (5)	1 (2)

DMT: Disease Modifying Therapy, VAS: Visual Analogue Scale.

*Language survey completed in.

Percentages were rounded to the largest integer.

Table 3 presents for the development sample the mean RS and SG values for level 2 and level 3 of each item in the PBMSI as well as two multi-attribute health states, all at level 2 and all at level 3. All health states were rated lower using the RS than the SG. The mean RS values ranged from 0.20 to 0.65, whereas the mean SG values ranged from 0.60 to 0.91. Also presented are the correlation coefficients between the RS and SG; weak correlations were observed ranging from -0.29 to 0.15.

**Table 3:** Mean rating scale and standard gamble values derived from the development sample.

Item and level	RS*	SG*	Correlation
	Mean (SD)	Mean (SD)	Coefficient
**Walking**			
Intermediate	0.65 (0.22)	0.87 (0.24)	0.07
Worst	0.49 (0.24)	0.82 (0.24)	0.11
**Fatigue**			
Intermediate	0.62 (0.19)	0.89 (0.21)	-0.09
Worst	0.46 (0.22)	0.81 (0.25)	-0.11
**Mood**			
Intermediate	0.62 (0.19)	0.90 (0.20)	0.15
Worst	0.46 (0.28)	0.84 (0.22)	-0.29
**Concentration**			
Intermediate	0.64 (0.20)	0.91 (0.19)	0.13
Worst	0.53 (0.22)	0.88 (0.21)	-0.006
**Roles & Responsibilities**			
Intermediate	0.65 (0.22)	0.87 (0.20)	0.09
Worst	0.39 (0.23)	0.80 (0.22)	0.18
**All intermediate**	0.48 (0.20)	0.84 (0.20)	0.12
**All worst**	0.20 (0.22)	0.60 (0.28)	0.07

*VAS values were measured on a worst imaginable-best imaginable scale, SG utilities were measured on a dead-perfect health scale.

**Fig 2:**
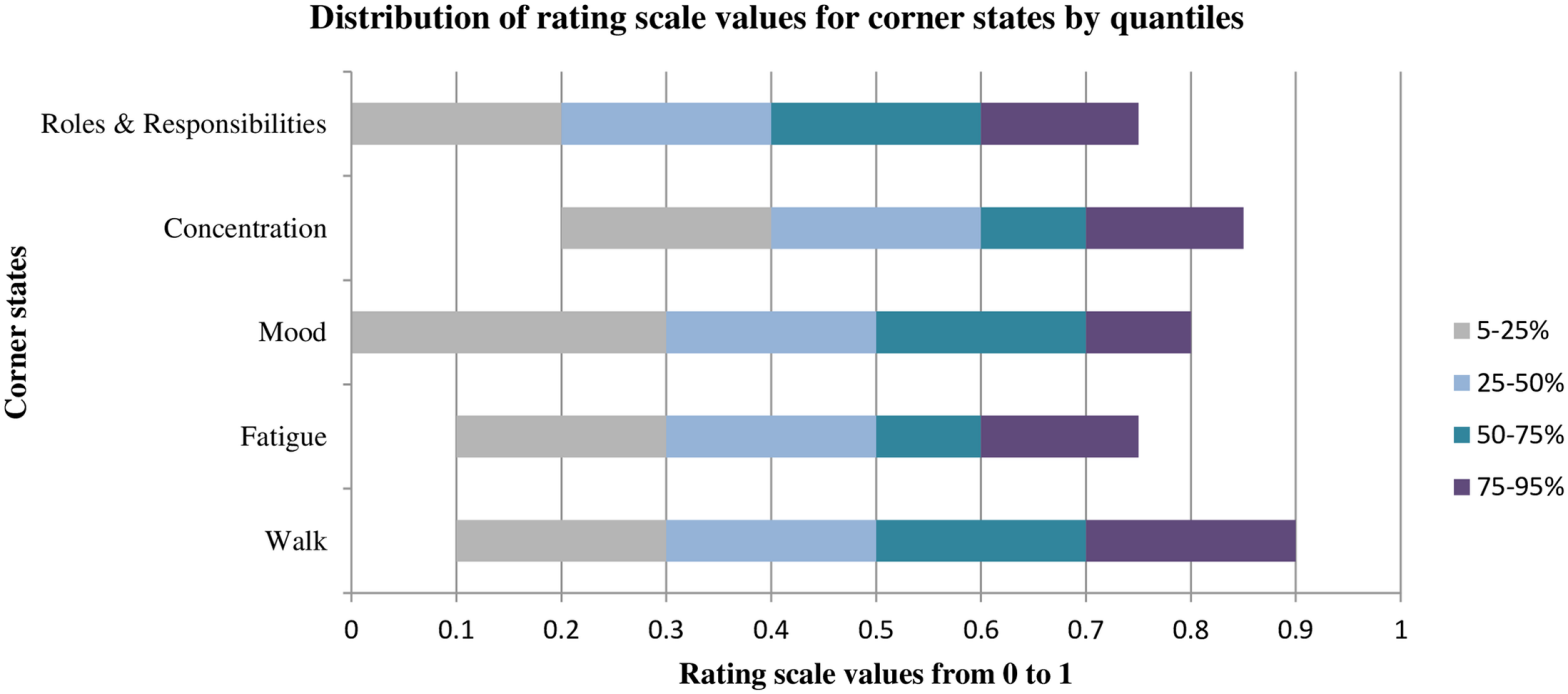
RS values by quantiles for PBMSI corner states in the development sample.

Fig 2 presents a distribution of the RS values by percentile for each of the corner states (i.e. level 3, worst, for each item). Higher scores on the RS indicate better health. The RS values were fairly evenly distributed. The median value, which is represented by the end of the light gray bar, was 0.5 for severe walking impairment, severe fatigue and depression. The median value for impaired concentration and restricted roles and responsibilities were 0.6 and 0.4, respectively.

**Fig 3:**
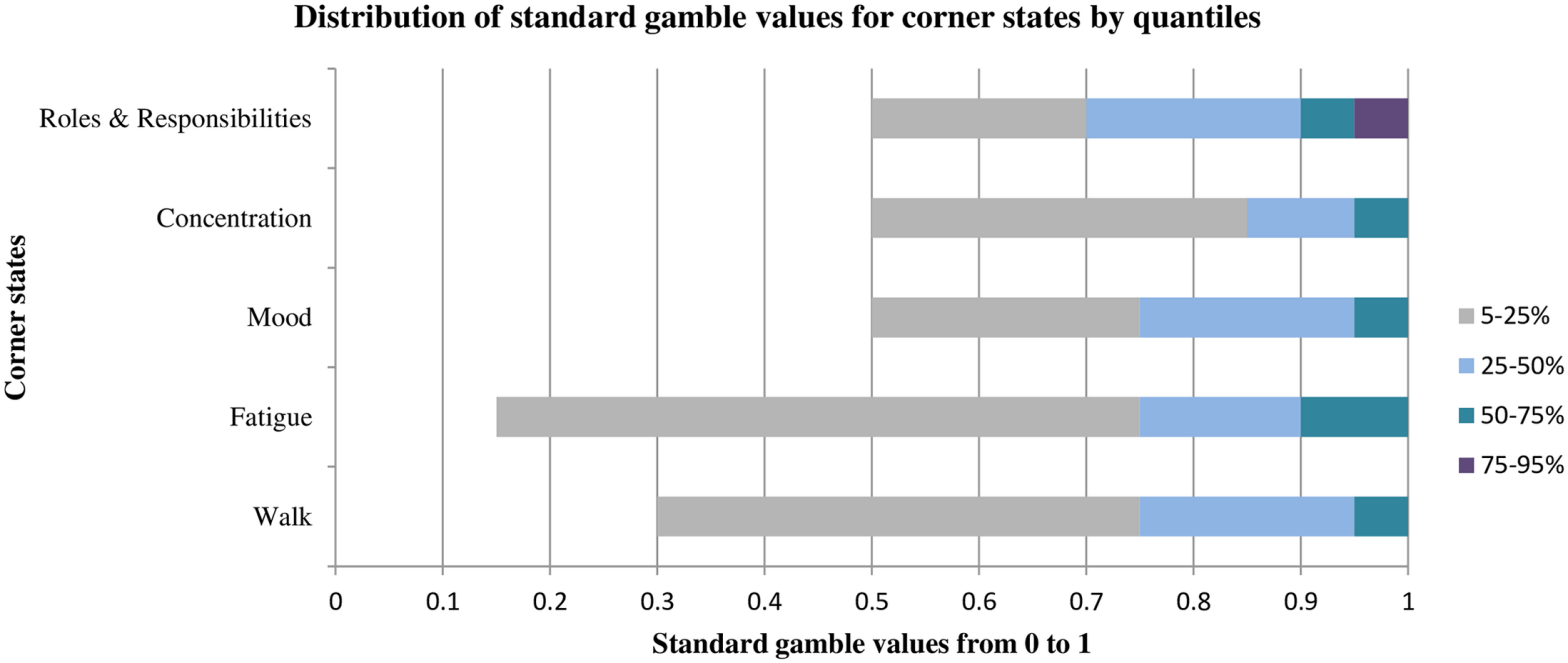
SG values by quantiles for PBMSI corner states in the development sample.

Fig 3 presents the percentile distribution of the SG values for the corner states. The SG values were on a scale from 0 to 1, where higher scores indicate better health. The SG values were considerably higher than RS values for all of the items, with the median values being 0.9 or 0.95. Twenty-five percent of the sample rated having severe walking impairments, severe fatigue, and severe impaired concentration equivalent to perfect health (1.0).

Table 4 presents the percent agreement between the levels of difficulty reported by patients for the SG (rows) and RS (columns). Across all levels of difficulty, 38% (23/61) found both methods to be of equal difficulty (diagonal cells); 50% (30/61) rated the SG at a higher level of difficulty than the RS (cells below the diagonal). Only 5 people rated the RS harder than the SG (cells above the diagonal), but the 6 people rating SG as “very easy” scored all health states with virtually the same value, 0.95 (data not shown). Chance corrected agreement as estimated using un-weighted Kappa was poor (k 0.09; 95% CI: 0.08 to 0.25) and poor using weighted Kappa (k 0.13; (95% CI: -0.08 to 0.34).

**Table 4:** Concordance between the levels of difficulty between the RS and the SG in the development sample.

Standard Gamble	Rating Scale				
	Very easy	Fairly easy	Fairly difficult	Very difficult	Total
**Very easy**	3 (5%)	2 (3%)	1 (2%)	0 (0%)	6 (10%)
**Fairly easy**	2 (3%)	12 (20%)	4 (7%)	1 (2%)	19 (31%)
**Fairly difficult**	1 (2%)	16 (26%)	8 (13%)	0 (0%)	25 (41%)
**Very difficult**	1 (2%)	3 (5%)	7 (11%)	0 (0%)	11 (18%)
**TOTAL**	7 (12%)	33 (54%)	20 (33%)	1 (2%)	61 (100%)

RS: Rating Scale; SG: Standard Gamble

Simple Kappa: 0.09 (95%CI -0.08 to 0.25); Weighted Kappa: 0.13 (-0.08 to 0.34)

To answer the question as to whether level of difficulty had an impact on health state values, we regressed method of valuation (SG, RS), on the 12 health state values, using generalized estimating equations which considered the correlation (non-independence) of the valuation, including the interaction between method and health state. The model was health state value = method (RS/SG) + item (1–12) + method*item. As the interaction term was non-significant, it was dropped. For the RS, the effect of difficulty across all items when compared to the SG was equal to -0.25. When the model was adjusted for difficulty, the difference was accentuated to -0.32. The difference between RS and SG did not depend on item (non-significant interaction).

Table 5 presents the parameters used to develop the MAUF_D_ based on the RS and SG values obtained in the development sample. The first column presents the mean RS and SG utility values for each response level, where level 1 was the best, level 2 was intermediate, and level 3 was the worst. The first level of each item was 1.0 (perfect health). As expected, there was a drop in utility values from level 1 to level 2 to level 3. For each item, response level 3 was the corner state utility value. The second column of Table 4 presents the disutility values (1-utility) for each of the item response levels. The third column presents the mean utility values rescaled so that the third response level of each item was 0.0, and the first response level was 1.0. The fourth column is the rescaled mean disutility score, which is equal to 1—the rescaled mean utility score (presented in third column). These are the parameters used to develop valuation function (MAUF_D_).

**Table 5:** Calculation of parameters in the estimation of the PBMSI MAUF_D_ in the Development sample.

Item & level	Mean utility		Mean disutility		Rescaled mean utility ^a^		Rescaled mean disutility ^b^	
	RS	SG	RS	SG	RS	SG	RS	SG
**Walking**								
**1**	1.00	1.00	0.00	0.00	1.00	1.00	0.00	0.00
**2**	0.53	0.87	0.47	0.13	0.29	0.28	0.71	0.72
**3**^c^	0.33	0.82	0.67	0.18	0.00	0.00	1.00	1.00
**Fatigue**								
**1**	1.00	1.00	0.00	0.00	1.00	1.00	0.00	0.00
**2**	0.48	0.89	0.52	0.11	0.25	0.42	0.75	0.58
**3**^c^	0.31	0.81	0.69	0.19	0.00	0.00	1.00	1.00
**Mood**								
**1**	1.00	1.00	0.00	0.00	1.00	1.00	0.00	0.00
**2**	0.46	0.90	0.54	0.10	0.31	0.38	0.69	0.63
**3**^c^	0.22	0.84	0.78	0.16	0.00	0.00	1.00	1.00
**Concentration**								
**1**	1.00	1.00	0.00	0.00	1.00	1.00	0.00	0.00
**2**	0.49	0.91	0.51	0.09	0.26	0.25	0.74	0.75
**3**^c^	0.31	0.88	0.69	0.12	0.00	0.00	1.00	1.00
**Roles & Responsibilities**								
**1**	1.00	1.00	0.00	0.00	1.00	1.00	0.00	0.00
**2**	0.43	0.87	0.57	0.13	0.30	0.35	0.70	0.65
**3**^c^	0.18	0.80	0.82	0.20	0.00	0.00	1.00	1.00

^a^ Rescaled mean utility score = (person mean utility score Level X–person mean utility score Level 3) / (person mean utility score Level1—person mean utility score Level3)

^b^ Rescaled mean disutility score = 1 –(rescaled utility score)

^c^ Corner states

Table 6 presents the MAUF_D_ developed using the SG values presented in Table 4. The sum of the corner states was equal to 0.85, which is less than 1.0, therefore the MAUF_D_ was multiplicative and yielded two solutions for Eq 2. Based on Eq 3c, the non-trivial solution was greater than 0. Using the iterative solution (Eq 2) an exact value for the non-trivial solution *c* was calculated, and found to be equal to 0.4821.

**Table 6:** PBMSI MAUF_D_ developed based on standard gamble values obtained from the Development sample.

Walking		Fatigue		Mood		Concentration		Roles & Responsibilities	
Level	*u*’_1_	Level	*u*’_2_	Level	*u*’_3_	Level	*u*’_4_	Level	*u*’_5_
**Single attribute mean disutilities**									
1	0.00	1	0.00	1	0.00	1	0.00	1	0.00
2	0.72	2	0.58	2	0.63	2	0.75	2	0.65
3	1.00	3	1.00	3	1.00	3	1.00	3	1.00
**Scaling parameter and corner state disutilities**									
*c* =	0.4821	*c*_*1*_ =	0.18	*c*_*3*_ =	0.16	*c*_*5*_ =	0.20		
		*c*_*2*_ =	0.19	*c*_*4*_ =	0.12				
**Valuation function**									
*PBMSI disutility*_(perfect health = 0, dead = 1)_ = (1/0.4821) x ([1 + {0.4821} x 0.18 x *u*’_1_] x ([1 + {0.4821} x 0.19 x *u*’_2_] x ([1 + {0.4821} x 0.16 x *u*’_3_] x ([1 + {0.4821} x 0.12 x *u*’_4_] x ([1 + {0.4821} x 0.20 x *u*’_5_]– 1)									
*PBMSI utility*_(dead = 0, perfect health = 1)_ = 1 –*PBMSI disutility*_(perfect health = 0, dead = 1)_									

The SG MAUF_D_ for the PBMSI in dis-utilities was:

PBMSIDisutility(perfecthealth=0;dead=1)=(10.4821)*([1+0.4821*0.18*u1,]*[1+0.4821*0.19*u2,]*[1+0.4821*0.16*u3,]*[1+0.4821*0.12*u4,]*[1+0.4821*0.20*u5,]−1)

Where the values of *u*’_1_, *u*’_2_, *u*’_3_, *u*’_4_, *u*’_5_ (the single-attribute mean disutilites) are selected from Table 7 depending on the individual’s responses to the PBMSI items. The calculated disutility on the perfect health = 0.0, dead = 1.0 scale can then be converted into a utility score on a dead = 0.0, perfect health = 1.0 scale:

$$PBMSIutility_{\left(\text{dead }=\;0,\text{ perfect health }=\text{1}\right)}=\text{ 1 – PBMSI disutility}$$

**Table 7:** PBMSI MAUF_D_ developed based on rating scale values obtained from the Development sample.

Walking		Fatigue		Mood		Concentration		Roles & Responsibilities	
Level	*u*’_1_	Level	*u*’_2_	Level	*u*’_3_	Level	*u*’_4_	Level	*u*’_5_
**Single attribute mean disutilities**									
1	0.00	1	0.00	1	0.00	1	0.00	1	0.00
2	0.71	2	0.75	2	0.69	2	0.74	2	0.70
3	1.00	3	1.00	3	1.00	3	1.00	3	1.00
**Scaling parameter and corner state disutilities**									
*c* =	-0.9987	*c*_*1*_ =	0.67	*c*_*3*_ =	0.78	*c*_*5*_ =	0.82		
		*c*_*2*_ =	0.69	*c*_*4*_ =	0.69				
**Valuation function**									
*PBMSI disutility*_(perfect health = 0, dead = 1)_ = (1/-0.9987) x ([1 + {-0.9987} x 0.67 x *u*’_1_] x([1 + {-0.9987} x 0.69 x *u*’_2_] x ([1 + {-0.9987} x 0.78 x *u*’_3_] x ([1 + {-0.9987} x 0.69 x *u*’_4_] x ([1 + {-0.9987} x 0.82 x *u*’_5_]– 1)									
*PBMSI utility*_(dead = 0, perfect health = 1)_ = 1 –*PBMSI disutility*_(perfect health = 0, dead = 1)_									

Table 7 presents the MAUF_D_ based on the RS values. The procedure used to develop the RS MAUF_D_ was identical to the process described for the SG MAUF_D_. Using the RS values, the sum of the corner states was equal to 3.65 and the scaling parameter was calculated to be equal to -0.9987. The full valuation function can be found in Table 7.

**Fig 4:**
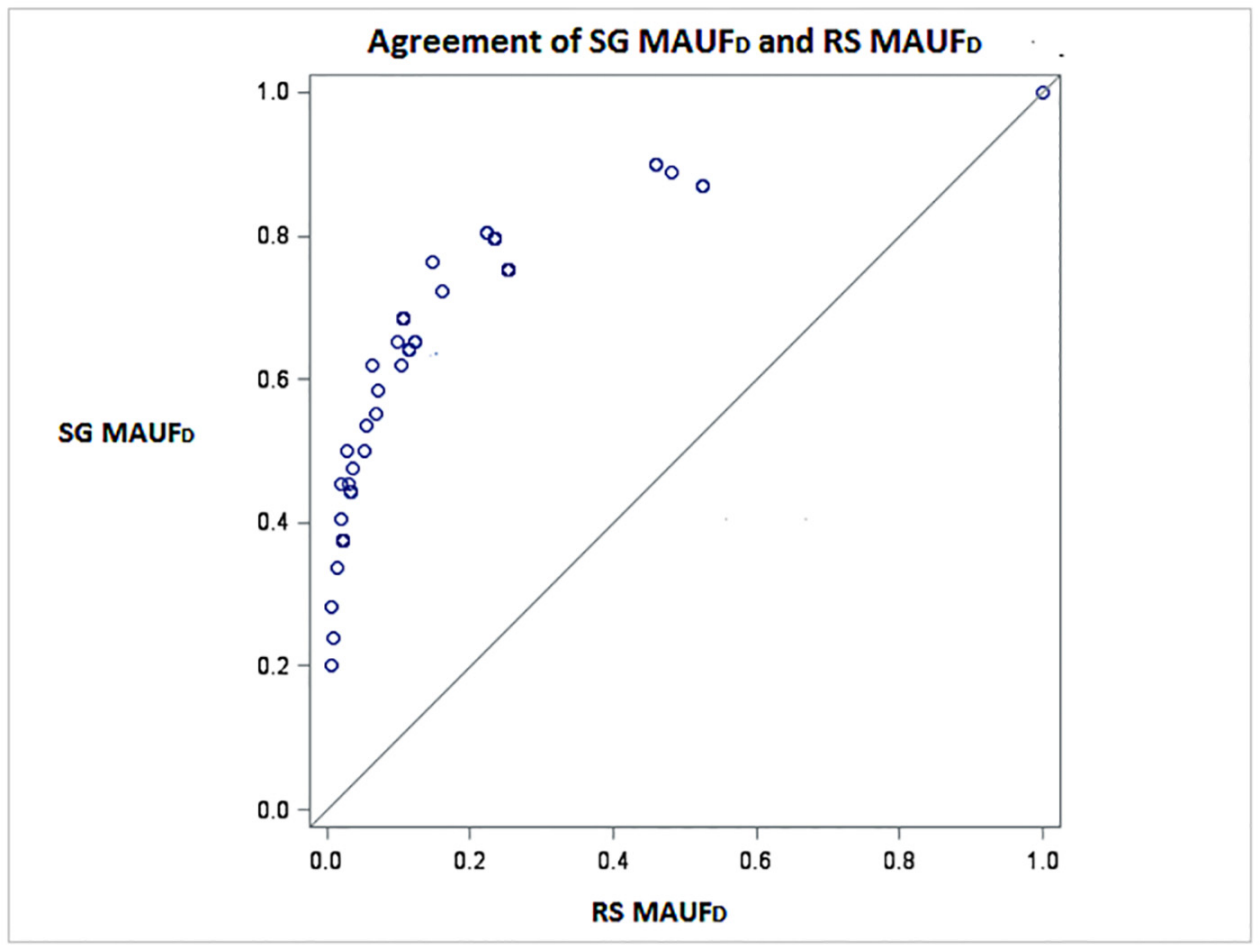
Scatter plot to assess agreement between the SG MAUF_D_ and the RS MAUF_D_ for the development sample.

Fig 4 presents, for the development sample, a scatter plot to assess agreement between PBMSI scores obtained using the RS MAUF_D_ against scores obtained using the SG MAUF_D_. As none of the data points were on the line of equality there was no agreement between the two methods. Scores produced by SG MAUF_D_ were consistently considerably higher than scores produced by the RS MAUF_D_, yielding a strong correlation (0.8), but no agreement.

**Fig 5:**
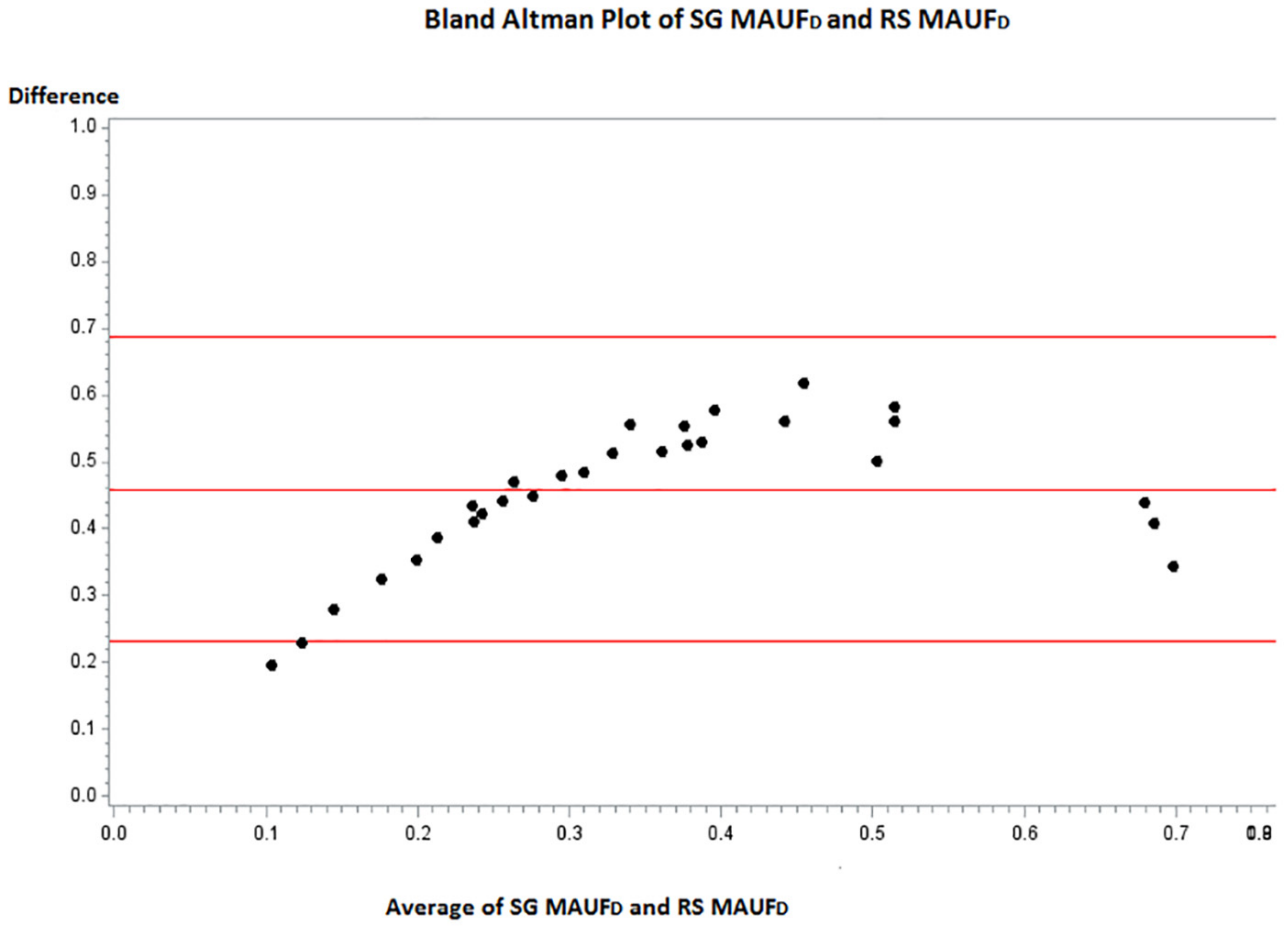
Bland-Altman plot to assess agreement between the SG MAUF_D_ and the RS MAUF_D_ in the development sample.

Fig 5 presents, for the development sample, the Bland-Altman plot between the SG MAUF_D_ and the RS MAUF_D_. The *x* axis shows the mean of the results of the two methods ([SG MAUF_D_ + RS MAUF_D_)/2), which is considered to represent the latent trait of “utility”. The *y* axis is the absolute difference between the two methods ([SG MAUF_D_ − RS MAUF_D_)). If the methods are concordant, the mean difference should be 0 with no pattern across the latent trait. The average difference between the methods was 0.46 (represented by the middle red line), and 95% of patients had a difference in scores between 0.24 and 0.68. A clinically meaningful difference on the SG or RS is 0.10; therefore the mean difference between the two methods was almost 5 times greater than the clinically meaningful difference. Additionally, there was a distinct pattern to the values such that, at the low end of the latent trait (poor health state) the differences were small; as latent health state improved, the difference between the methods increased. A paired t-test revealed that the difference in scores was statistically significant (p-value <0.0001).

**Fig 6:**
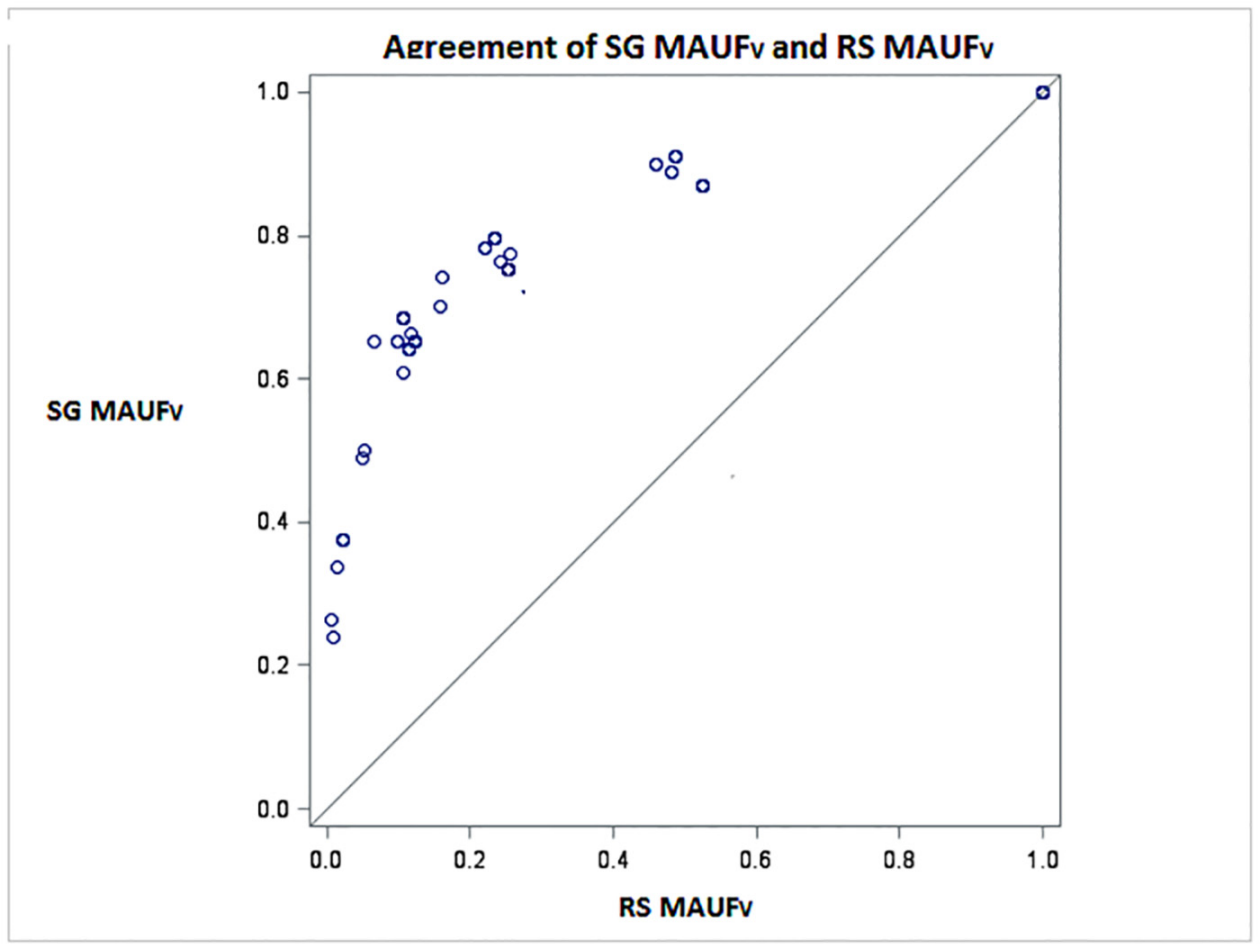
Scatter plot to assess agreement between the SG MAUF_V_ and the RS MAUF_V_ for the validation sample.

Fig 6 presents, for the validation sample, a scatter plot of the PBMSI scores obtained using the RS MAUF_V_ against scores obtained using the SG MAUF_V_. Similar to the results obtained for the development sample, there was no agreement between scores produced by the two MAUF_V_.

**Fig 7:**
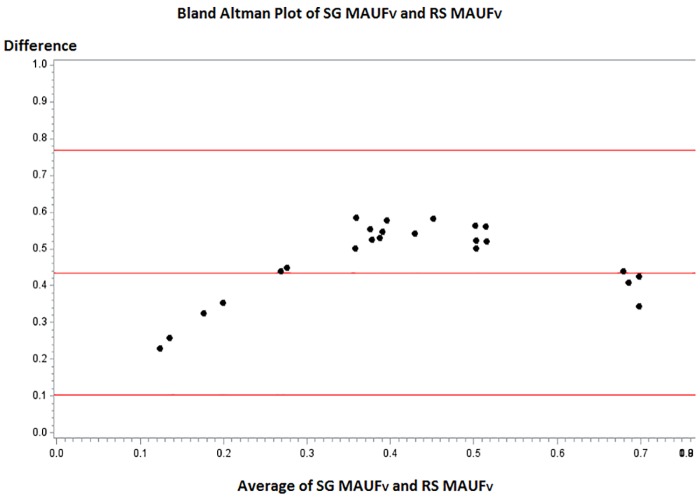
Bland-Altman plot to assess agreement between the SG MAUF_V_ and the RS MAUF_V_ in the validation sample.

Fig 7 presents the Bland Altman plot for the validation sample, which shows that the mean difference between the SG MAUF_V_ and RS MAUF_V_ is 0.44, 4 times greater than the clinically meaningful difference of 0.1 points. A paired t-test between scores indicated that the difference in scores between the SG MAUF_V_ and RS MAUF_V_ was statistically significant (p-value <0.0001).

Table 8 presents for the validation sample, the ability of the SG and RS MAUF_V_ to discriminate between different clinical subgroups, assessed using the PDDS, 6MWT and the general health perception item of the RAND-36. Both the SG MAUF_V_ and the RS MAUF_V_ were able to differentiate between different levels of disability measured using the PDDS. However, the RS MAUF_V_ had a wider range of values than the SG MAUF_V_. The EQ-5D valuation function was not able to differentiate between moderate and severe levels of disability. For the 6MWT, both the SG and the RS MAUF_V_ were able to differentiate between different levels of walking capacity, however, the values produced by the RS MAUF_V_ were lower than the SG MAUF_V_. The EQ-5D was also able to differentiate between different levels of walking capacity. As for general health perception, the SG MAUF_V_ was able to differentiate between all levels of health perception. However, the RS MAUF_V_ was only able to differentiate between excellent, very good and good health, but not between good and fair health. The EQ-5D also presented with problems discriminating between different levels of health perception, specifically between very good and good.

**Table 8:** Known-groups validity of the PBMSI and the EQ-5D index against external measures of disease severity in the validation sample.

Measure	SG MAUF_V_	RS MAUF_V_	EQ-5D
	Mean (SD)	Mean (SD)	Mean (SD)
**PDDS**			
0–1 (mild)	0.79 (0.15)*	0.63 (0.41)*	0.77 (0.08)*
2–3 (moderate)	0.67 (0.19)	0.23 (0.19)	0.66 (0.12)
4–5 (severe)	0.58 (0.23)	0.10 (0.08)	0.69 (0.12)
**6MWT**			
600 + m	0.89 (0.14)*	0.38 (0.38)*	0.78 (0.08)*
300 to 599m	0.70 (0.17)	0.22 (0.18)	0.71 (0.12)
0 to 299m	0.53 (0.25)	0.12 (0.10)	0.50 (0.20)
**General Health Perception**			
Excellent	0.88 (0.21)*	0.71 (0.51)*	0.77 (0.15)
Very Good	0.79 (0.15)	0.36 (0.31)	0.73 (0.13)
Good	0.70 (0.16)	0.21 (0.15)	0.72 (0.12)
Fair	0.62 (0.31)	0.31 (0.46)	0.59 (0.12)
Poor	---	---	---

PDDS, Patient Determined Disease Steps; 6MWT, 6-Minute Walk Test; PBMSI, Preference-Based Multiple Sclerosis Index; m, Meters; SD, Standard Deviation.

*Linear test for trend, p-value < 0.05

## Discussion

To fill a gap in outcome measurement for interventions targeting HRQL, this study elicited patient preferences for items in a new measure, the PBMSI, using two standard methods, the SG and the RS. A MAUF was developed based on values obtained using each of the methods, and the validity of these scoring algorithms were tested in a separate sample of MS patients (i.e. validation sample). In contemplating trade-offs in the selection of a preference-based elicitation approach for a MAUF that could guide clinical decision making, results suggest the RS is preferable in terms of feasibility and validity for MS patients.

The SG and the RS produced considerably different results from each other. On a scale from 0 (dead) to 1 (perfect health), values produced by the SG were consistently higher than those produced by the RS. The median values for the corner state items were between 0.4 and 0.6 on the RS, and between 0.90 and 0.95 on the SG. With the SG, 50% of the sample rated having severe walking impairments, severe fatigue, severe impaired concentration and depression close or equivalent to perfect health (1.0). For these same items, none of the respondents gave a value of 1.0 on the RS.

Our results are similar to previous studies that have compared the SG and the RS. Jansen and colleagues[29] compared the two methods in 51 women with breast cancer. They asked patients to value a hypothetical chemotherapy scenario, and reported that values elicited using the SG (mean ~0.9) were consistently higher than the RS (mean ~0.6). Juniper and colleagues[30] compared the SG and RS in 40 patients with asthma. In their study, more than half of the patients (n = 23) rated their current health equal to 1.0 (perfect health) on the SG, even though they represented patients at the more severe end of the spectrum (80% required inhaled steroids). Sullivan and colleagues[31] interviewed 52 patients with diabetes mellitus on various health states describing different levels of disease severity in diabetic peripheral neuropathy. The SG scores were considerably higher than the RS. The highest median preference score for the SG was 0.96 (mild neuropathy) and the lowest was 0.65 (below-knee amputation). On the RS, the highest median score was 0.89 (mild neuropathy) and the lowest was 0.23 (below-knee amputation).

In our study, correlations between the SG and the RS were very weak (r ~ 0.1), thus reinforcing the fact that there were considerable discrepancies in the values elicited by the two methods. These low correlations were similar to what others have reported in cancer (r = 0.18),[29] chronic musculoskeletal pain (r = 0.21)[32] and asthma(r = 0.18).[33]

The SG is a method that assesses the probability an individual would risk death to regain perfect health. As death is a highly undesirable state, patients may be inclined to stop the gambling earlier, thus resulting in an overestimate of the value associated with an impaired health state.[17, 34] In the context of MS, the possible risk of dying after treatment is far from realistic as existing medical treatments are rarely life threatening. Instead treatment is directed at slowing the progression of disease or disability. As the RS does not involve risk or decision making under uncertainty, values elicited with this method tend to be systematically lower than the SG.

Fifty percent of our sample rated the SG at a higher level of difficulty than the RS. These findings are concordant with previous studies that have compared the SG with the RS. In patients with cancer, Dobrez and Calhoun[35] reported that 17% of their sample did not comprehend the SG method. Similarly in HIV/AIDS patients, Sakthong[36] and colleagues reported that the SG was more difficult for patients to understand compared to the RS (p = 0.002), and that the completion time for the SG was much longer than the RS (average 5 minutes per health state vs 0.9 minutes per health state).

The SG method may be difficult for patients to comprehend because the concept of probabilities is a challenging one to grasp and far from everyday experience.[37] Lack of comprehension of the method is an important issue in the valuation of health states, as it can compromise the accuracy or reliability of the data collected.[38]

There were several notable features of this study. First, we used an internet based approach to value health states, rather than the traditional interviewer based approach. Traditionally the SG requires the use of a trained interviewer with props, where researchers must either go to the participant’s home or offer sufficient incentives to bring the participant to the lab, which are both expensive. The advantage of using an online survey is that patients can complete the survey in the convenience of their home, resulting in greater recruitment or participation. Although other studies have used the internet to elicit preferences,[39, 40] the validity and reliability of this approach requires further study. Second, in the SG, rather than alternating the proportion of success and death in a “ping-pong” manner we simply asked individuals to indicate the maximum risk of death they were willing to take with the hypothetical treatment. This may have resulted in a higher value of utilities than the former approach. Finally, alternate methods of valuation such as the time trade-off (number of years patients are willing to trade off for perfect health) were not assessed in this study.

## Conclusions

This study elicited patient preferences for various items from a MS-specific classification system using two different valuation methods, the SG and RS. We compared these two methods in terms of the values they produced, their difficulty of use and impact on the MAUF. Our findings demonstrated that, the SG compared to the RS, produced higher utility and was more difficult for patients to understand. Although RS is considered inappropriate as a basis for obtaining Quality Adjusted Life Years (QALYs) for economic evaluation, in contemplating trade-offs in the selection of a preference-based elicitation approach for a MAUF that could guide clinical decision making, results suggest the RS is preferable in terms of feasibility and validity for MS patients.

Furthermore, in the broader policy arena of allocating resources across multiple health conditions, the standard approach of using generic preference-based measures with general population weights would be difficult to disapprove. However, in the context of use here, which would be to evaluate the effect of interventions that are expected to impact widely on the health of individuals with MS, the PBMSI with patient preferences shows promise.

## Abbreviations

MS: Multiple sclerosis
SG: standard gamble
RS: rating scale
MAUF: multi-attribute utility function
MAUF_D_: multi-attribute utility function development sample
MAUF_V_: multi-attribute utility function validation sample
QALYs: Quality Adjusted Life Years
PBMSI: Preference-Based Multiple Sclerosis Index
PDDS: Patient-Determined Disease Steps
EDSS: Expanded Disability Status Scale
SD: standard deviation

## References

[1] Kuspinar A, Rodriguez AM, & Mayo NE. The effects of clinical interventions on health-related quality of life in multiple sclerosis: a meta-analysis. Multiple Sclerosis Journal 2012; 18: 1686–1704. 10.1177/1352458512445201 23235779

[2] Ware John E Jr, Sherbourne CE: The MOS 36-item short-form health survey (SF-36): I. Conceptual framework and item selection. Medical Care 1992; 473–483. 1593914

[3] Freeman JA, Hobart JC, and Thompson AJ. Does adding MS-specific items to a generic measure (the SF-36) improve measurement? *Neurology* 57 1 (2001): 68–74. 11445630 10.1212/wnl.57.1.68

[4] Shaw JW, Johnson JA, Coons SJ. US valuation of the EQ-5D health states: development and testing of the D1 valuation model. Medical care 2005; 43: 203–220. 15725977 10.1097/00005650-200503000-00003

[5] Feeny D, Furlong W, Torrance GW, Goldsmith CH, Zhu Z, DePauw S, et al Multiattribute and single‐attribute utility functions for the health utilities index mark 3 system. Medical care 2002;40: 113–128. 11802084 10.1097/00005650-200202000-00006

[6] Peeters Y, Stiggelbout AM: Health state valuations of patients and the general public analytically compared: a meta-analytical comparison of patient and population health state utilities. Value Health 2010;13:306–309. 10.1111/j.1524-4733.2009.00610.x 19744288

[7] Kuspinar A, Mayo NE: A review of the psychometric properties of generic utility measures in multiple sclerosis. Pharmacoeconomics 2014;32:759–773. 10.1007/s40273-014-0167-5 24846760

[8] Bennett KJ, Torrance GW: Measuring health state preferences and utilities: rating scale, time trade-off, and standard gamble technqiues; in Spilker B (ed): Quality of Life and Pharmaeconomics in Clinical Trials. Philadelphia, Lippincott-Raven Publishers, 1996, pp 253–267.

[9] Kuspinar A, Mayo NE: Do generic utility measures capture what is important to the quality of life of people with multiple sclerosis? Health Qual Life Outcomes 2013;11:71 10.1186/1477-7525-11-71 23618072 PMC3649951

[10] Kuspinar A, Finch L, Pickard S, Mayo NE: Using existing data to identify candidate items for a health state classification system in multiple sclerosis. Qual Life Res 2014;23:1445–1457. 10.1007/s11136-013-0604-5 24338161

[11] Kuspinar A, Bouchard V, Moriello C, & Mayo NE. The Development of a Bilingual MS-Specific Health Classification System: The Preference-Based Multiple Sclerosis Index (PBMSI). *International Journal of MS Care* 2015 (online first). 10.7224/1537-2073.2014-106 PMC4849398 27134579

[12] Mayo NE, Bayley M, Duquette P, Lapierre Y, Anderson R, Bartlett S: The role of exercise in modifying outcomes for people with multiple sclerosis: a randomized trial. BMC neurology 2013;13:69 10.1186/1471-2377-13-69 23809312 PMC3706216

[13] Gudex C. Standard Gamble user manual: props and self-completion methods. 1994.

[14] Kontodimopoulos N, Niakas D: Overcoming inherent problems of preference-based techniques for measuring health benefits: an empirical study in the context of kidney transplantation. BMC health services research 2006;6:3 16412242 10.1186/1472-6963-6-3 PMC1373617

[15] Oliver A: Testing the internal consistency of the standard gamble in success and failure frames. Social science & medicine 2004;58:2219–2229. 15047079 10.1016/j.socscimed.2003.08.024

[16] Dolan P, Sutton M: Mapping visual analogue scale health state valuations onto standard gamble and time trade-off values. Soc Sci Med 1997;44:1519–1530. 9160441 10.1016/s0277-9536(96)00271-7

[17] Robinson A, Loomes G, Jones-Lee M: Visual analog scales, standard gambles, and relative risk aversion. Medical Decision Making 2001;21:17–27. 11206943 10.1177/0272989X0102100103

[18] Learmonth YC, Motl RW, Sandroff BM, Pula JH, Cadavid D: Validation of patient determined disease steps (PDDS) scale scores in persons with multiple sclerosis. BMC Neurol 2013;13:37 10.1186/1471-2377-13-37 23617555 PMC3651716

[19] Marrie RA, Cutter G, Tyry T, Vollmer T, Campagnolo D: Does multiple sclerosis-associated disability differ between races? Neurology 25-4-2006;66:1235–1240. 16636241 10.1212/01.wnl.0000208505.81912.82

[20] Hohol MJ, Orav EJ, Weiner HL: Disease steps in multiple sclerosis: a longitudinal study comparing disease steps and EDSS to evaluate disease progression. Mult Scler 1999;5:349–354. 10516779 10.1177/135245859900500508

[21] Paltamaa J, West H, Sarasoja T, Wikstrom J, Malkia E: Reliability of physical functioning measures in ambulatory subjects with MS. Physiother Res Int 2005;10:93–109. 16146327 10.1002/pri.30

[22] Hays RD. RAND-36 health status inventory. San Antonio, CA: Psychological Corporation; 1998.

[23] Freeman JA, Hobart JC, Langdon DW, Thompson AJ: Clinical appropriateness: a key factor in outcome measure selection: the 36 item short form health survey in multiple sclerosis. J Neurol Neurosurg Psychiatry 2000;68:150–156. 10644779 10.1136/jnnp.68.2.150 PMC1736771

[24] Nortvedt MW, Riise T, Myhr KM, Nyland HI: Quality of life as a predictor for change in disability in MS. Neurology 12-7-2000;55:51–54. 10891905 10.1212/wnl.55.1.51

[25] Kind P: The EuroQol instrument: an index of health-related quality of life; in Spilker B (ed): Quality of Life and Pharmaeconomics in Clinical Trials. Philadelphia, Lippincott-Raven Publishers, 1996, pp 191–201.

[26] Furlong W, Feeny D, Torrance G, Goldsmith C, DePauw S, Zhu Z, et al. Multiplicative multi-attribute utility function for the Health Utilities Index Mark 3 (HUI3) system: a technical report. 1998. Centre for Health Economics and Policy Analysis (CHEPA), McMaster University, Hamilton, Canada.

[27] Ryan M, Gerard K: Discrete choice experiments; in Fayers P, Hays D (eds): Assessing quality of life in clincal trials. New York, Oxford University Press, 2005, pp 431–445.

[28] Shahrbanian S, Duquette P, Kuspinar A, Mayo NE: Contribution of symptom clusters to multiple sclerosis consequences. Qual Life Res 17-9-2014. 10.1007/s11136-014-0804-7 25228080

[29] Jansen SJ, Kievit J, Nooij MA, Stiggelbout AM: Stability of Patients Preferences for Chemotherapy The Impact of Experience. Medical Decision Making 2001;21:295–306. 11475386 10.1177/0272989X0102100405

[30] Juniper EF, Norman GR, Cox FM, Roberts JN: Comparison of the standard gamble, rating scale, AQLQ and SF-36 for measuring quality of life in asthma. European Respiratory Journal 2001;18:38–44. 11510803 10.1183/09031936.01.00088301

[31] Sullivan SD, Lew DP, Devine EB, Hakim Z, Reiber GE, Veenstra DL: Health state preference assessment in diabetic peripheral neuropathy. Pharmacoeconomics 2002;20:1079–1089. 12456202 10.2165/00019053-200220150-00004

[32] Goossens MlE, Vlaeyen JW, Rutten-van Molken MP, van der Linden SM: Patient utilities in chronic musculoskeletal pain: how useful is the standard gamble method? Pain 1999;80:365–375. 10204750 10.1016/s0304-3959(98)00232-2

[33] Blumenschein K, Johannesson M: Relationship between quality of life instruments, health state utilities, and willingness to pay in patients with asthma. Ann Allergy Asthma Immunol 1998;80:189–194. 9494453 10.1016/S1081-1206(10)62954-7

[34] Bleichrodt H: A new explanation for the difference between time trade-off utilities and standard gamble utilities. Health Econ 2002;11:447–456. 12112493 10.1002/hec.688

[35] Dobrez DG, Calhoun EA: Testing subject comprehension of utility questionnaires. Quality of Life Research 2004;13:369–376. 15085909 10.1023/B:QURE.0000018475.17665.6e

[36] Sakthong P, Schommer JC, Gross CR, Prasithsirikul W, Sakulbumrungsil R: Health utilities in patients with HIV/AIDS in Thailand. Value in Health 2009;12:377–384. 10.1111/j.1524-4733.2008.00440.x 20667064

[37] Lenert LA, Sturley AE. Use of the internet to study the utility values of the public. Proceedings of the AMIA Symposium, 440. 2002. American Medical Informatics Association. PMC2244417 12463862

[38] Wittenberg E, Prosser LA: Ordering errors, objections and invariance in utility survey responses. Applied health economics and health policy 2011;9:225–241. 10.2165/11590480-000000000-00000 21682351

[39] Stein K, Dyer M, Crabb T, Milne R, Round A, Ratcliffe J, et al: A pilot Internet "value of health" panel: recruitment, participation and compliance. Health and quality of life outcomes 2006;4. 10.1186/1477-7525-4-90 PMC1716763 17129380

[40] Chang WT, Collins ED, Kerrigan CL: An Internet-based utility assessment of breast hypertrophy. Plastic and reconstructive surgery 2001;108:370–377. 11496177 10.1097/00006534-200108000-00014

